# Identification of Physiological Substrates and Binding Partners of the Plant Mitochondrial Protease FTSH4 by the Trapping Approach

**DOI:** 10.3390/ijms18112455

**Published:** 2017-11-18

**Authors:** Magdalena Opalińska, Katarzyna Parys, Hanna Jańska

**Affiliations:** 1 Faculty of Biotechnology, University of Wroclaw, Fryderyka Joliot-Curie 14A, 50-383 Wroclaw, Poland; 2 Present address: Gregor Mendel Institute, Austrian Academy of Sciences, Vienna Biocenter, A-1030 Vienna, Austria; katarzyna.parys@gmi.oeaw.ac.at

**Keywords:** AAA protease, ATP-dependent proteolysis, mitochondria, inner mitochondrial membrane proteostasis, carbonylated proteins

## Abstract

Maintenance of functional mitochondria is vital for optimal cell performance and survival. This is accomplished by distinct mechanisms, of which preservation of mitochondrial protein homeostasis fulfills a pivotal role. In plants, inner membrane-embedded *i*-AAA protease, FTSH4, contributes to the mitochondrial proteome surveillance. Owing to the limited knowledge of FTSH4’s in vivo substrates, very little is known about the pathways and mechanisms directly controlled by this protease. Here, we applied substrate trapping coupled with mass spectrometry-based peptide identification in order to extend the list of FTSH4’s physiological substrates and interaction partners. Our analyses revealed, among several putative targets of FTSH4, novel (mitochondrial pyruvate carrier 4 (MPC4) and Pam18-2) and known (Tim17-2) substrates of this protease. Furthermore, we demonstrate that FTSH4 degrades oxidatively damaged proteins in mitochondria. Our report provides new insights into the function of FTSH4 in the maintenance of plant mitochondrial proteome.

## 1. Introduction

Mitochondria are life-essential multifunctional organelles. In addition to their vital role in energy conversion, mitochondria are involved in diverse metabolic pathways including iron sulfur cluster biosynthesis, in cellular signaling and in the regulation of programmed cell death. Owing to the central role of these organelles, mitochondrial dysfunction is implicated in the onset and pathology of a myriad diseases and aging [1,2,3]. Distinct quality control pathways are engaged in mitochondrial surveillance, maintaining functional mitochondria and facilitating adaptation to stress conditions [4,5,6]. Mitochondrial proteases play a central role in these mechanisms, not only by the removal of damaged proteins or excess subunits, but also as regulatory components. To fully understand the spectrum of processes that rely on the action of mitochondrial proteases, detailed knowledge of their physiological substrates and interaction partners is required. In this regard plant mitochondrial proteases, including *i*-AAA protease—FTSH4, still remain poorly characterized.

*i*-AAA protease forms a homo-oligomeric ATP-dependent proteolytic complex that is embedded in the inner mitochondrial membrane (IM) with the catalytic sites exposed to the intermembrane space (IMS) [7]. In humans, a homozygous mutation in the gene encoding *i*-AAA protease (YME1L) causes mitochondriopathy with optic nerve atrophy [8]. Identification of proteolytic substrates and binding partners of mammalian *i*-AAA protease revealed that YME1L by proteolytic processing of fusion factors, selective removal of misfolded and unassembled subunits and turnover of specific regulatory proteins, influences a myriad of processes inside the mitochondria [9,10,11].

The molecular function of the plant YME1L counterpart—FTSH4 is less understood. Thus far, only one physiological substrate of FTSH4 has been described. FTSH4 is required for the turnover of the essential subunit of the TIM23 translocase, Tim17-2, indicating FTSH4-dependent proteolytic regulation of pre-protein influx into the plant mitochondria [12]. However, under stress conditions, loss of FTSH4 is linked to broad pleiotropic phenotypes including oxidative stress, imbalance in phospholipid metabolism, perturbation in oxidative phosphorylation system activity and aberrant mitochondrial morphology, suggesting that plant *i*-AAA protease, like its mammalian homologue, controls distinct processes inside the mitochondria [13,14]. Overall, to fully understand the role of FTSH4 in the maintenance of functional mitochondria, the in-depth knowledge of its in vivo targets is necessary.

Here, we applied an unbiased substrate trapping approach in order to identify potential physiological substrates and biding partners of FTSH4. Our strategy is based on the identification of proteins co-purifying with a proteolytically inactive mutant of the protease. The trapping method was successfully used to search for substrates and interactors of the bacterial homolog of FTSH4, FtsH [15]. However, to date this method has not been employed for the analysis of plant mitochondrial proteases. In the present study, we found 17 potential candidates, among which we identified known FTSH4’s substrate, Tim17-2, and novel ones which are the subunit of the inner membrane translocase Pam18-2 and mitochondrial pyruvate transporter 4 (MPC4). Our analysis indicated that FTSH4 is predominantly linked to the mitochondrial protein import machinery, inner membrane organizing proteins and specific metabolic pathways. Furthermore, we provide evidence that oxidative protein damage might trigger FTSH4-dependent proteolysis.

## 2. Results and Discussion

### 2.1. Substrate Trapping Assay Reveals a List of Potential FTSH4 Targets and Its Interacting Partners inside Mitochondria

**Figure 1:**
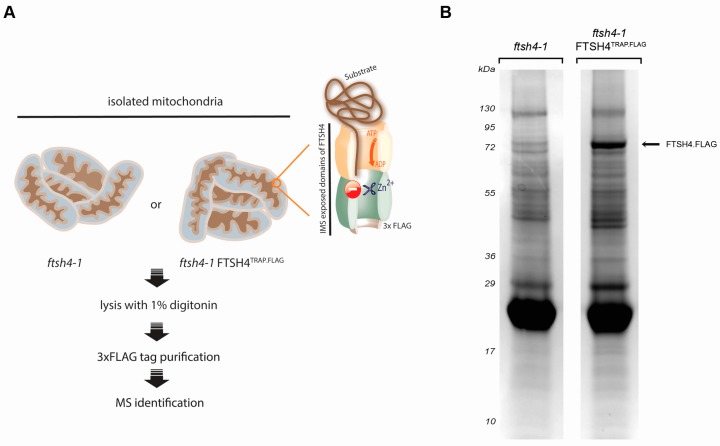
FTSH4 substrate trapping assay (**A**) Overview of FTSH4 substrate-trapping assay. Cartoon illustrating the experimental workflow; (**B**) Eluted fractions resolved on SDS-PAGE and stained with CBB. IMS—intermembrane space.

**Figure 2:**
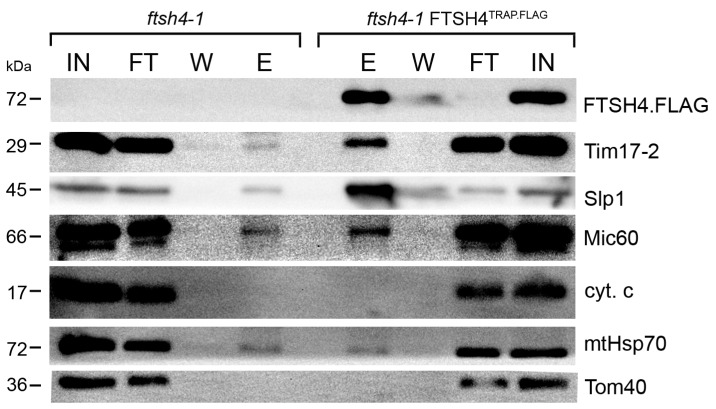
Immunoblot analysis of FTSH4 substrate trapping assay samples. Mitochondria from control (*ftsh4-1*) and *ftsh4-1* FTSH4^TRAP.FLAG^ were solubilized with digitonin and subjected to immunoprecipitation with anti-FLAG affinity matrix. The precipitated proteins were immunoblotted with antibodies against the indicated proteins. IN—input (5%), FT—flow-through (5%), W—Wash, E—eluate (50%).

The substrate trapping assay is a broadly used method enabling identification of enzyme substrates in an unbiased manner [15,16,17,18]. This experimental strategy has been applied to successfully identify physiological targets of diverse proteases, including membrane-bound bacterial FtsH [15]. Here, we employed this strategy in order to expand the spectrum of in vivo substrates and interaction partners of FTSH4. Briefly, we utilized an *Arabidopsis thaliana* line that produces, instead of functional wild type FTSH4, a proteolytically inactivated tagged version of this protease (*ftsh4-1* FTSH4^TRAP.FLAG^). Proteolytic activity of FTSH4 was abolished by a single point mutation in the catalytic center of the proteolytic chamber [12,15]. Since AAA domain of the FTSH4^TRAP.FLAG^ mutant is active, the substrates are translocated into the proteolytic chamber, where they are not degraded and thus remain trapped [15]. To characterize proteins caught inside the protease, affinity purification of FTSH4^TRAP.FLAG^ from mitochondria followed by mass spectrometry-based peptide identification was applied (Figure 1). Resulting proteins co-purifying with FTSH4^TRAP.FLAG^ in two independent biological replicates are listed in Table 1 and Table S1. Obtained data were validated by immunoblotting analyses with available antibodies (Figure 2).

The vast majority of identified proteins were localized to the inner mitochondrial membrane and were assigned to the four main functional classes according to Uniprot; transmembrane transport, metabolism, mitochondrial protein import and membrane organization (Table 1). This list includes potential interaction partners (e.g., adaptor proteins) and substrates of the FTSH4 protease inside mitochondria. Since yeast or mammalian *i*-AAA proteases degrade damaged or excessive subunits, transport intermediates (preproteins) and regulatory proteins [9,10,11,19], it is conceivable to find all these diverse types of substrates amongst proteins co-purifying with FTSH4^TRAP.FLAG^.

Several proteins identified in our assay reinforce the existence of a link between FTSH4 and mitochondrial ATP production by oxidative phosphorylation [14,20]. These include members of the mitochondrial substrate carrier family such as orthologues of mitochondrial NAD transporter (NDT2) [21] and mitochondrial pyruvate carrier (MPC4) [22]. In addition, amongst proteins related to mitochondria energy conversion that were found to co-purify with FTSH4^FLAG.TAG^, we also identified components of succinate dehydrogenase flavoprotein complex; subunit 1 (SDH1-1) and subunit 5 (SDH5). These are peripheral membrane proteins localized to the matrix side of the inner membrane [23]. Based on the finding concerning the human counterpart of FTSH4 protease—YME1L [19], matrix localized proteins like SDH1-1, SDH5 and isocitrate dehydrogenase NAD regulatory subunit 1 (IDH1) could be FTSH4 substrates during import of their precursors from the cytosol into the mitochondrial matrix. Interestingly, SDH1-1 specifically accumulates in plants devoid of functional FTSH4 (*ftsh4* mutants) [14]. Alternatively, observed interactions with these proteins could occur through the N-terminal domain of FTSH4 that protrudes into the matrix. In addition, we found an accessory subunit of the respiratory complex dihydroorotate dehydrogenase [24] and intermembrane space localized l-galactono-1,4-lactone dehydrogenase (GLDH) [25] as potential FTSH4 targets. In *Arabidopsis thaliana*, GLDH not only catalyzes the last step of ascorbate biosynthesis pathway, but it also has a second non-enzymatic function in the assembly of respiratory complex I [25].

Furthermore, our data indicate a connection between FTSH4 and mitochondrial protein biogenesis pathways. We found an essential subunit of TIM17:23 translocase, Tim17-2, among proteins co-purifying with FTSH4^TRAP.FLAG^. This protein was recently identified as a physiological substrate of the plant *i*-AAA protease [12]. FTSH4 protease regulates pre-protein influx into the mitochondria through the turnover of the Tim17-2 subunit. This function of *i*-AAA protease was also described in mammals, where YME1L controls mitochondrial protein import through the degradation of the Tim17A subunit, a homologue of Tim17-2 [10]. In addition, in the substrate trapping assay we found homologues of Pam18, an essential subunit of the pre-sequence translocase-associated (PAM) motor that is recruited by TIM23 translocase [26], and many other components involved in mitochondrial pre-protein uptake, implying the association of FTSH4 with these mitochondrial protein biogenesis machineries. 

Our list of potential FTSH4 targets also includes *A. thaliana* homologue of highly conserved inner membrane protein Mitofilin (also called Mic60 or Fcj1 (in yeast)). Mic60 is a central component of the MICOS complex (mitochondrial contact site complex) that is essential for cristae junction formation [27]. Mammalian *i*-AAA protease, YME1L regulates Mic60/Mitofilin homeostasis, which is required for the maintenance of mitochondrial morphology and organization of mtDNA nucleoids. Impaired assembly of the MICOS complex is associated with the formation of giant mitochondria due to the dysregulated fusion and fission events [28]. Interestingly, also in *Arabidopsis* disturbance in Mic60 levels results in the formation of giant mitochondria [29]. Strikingly, similar mitochondrial morphology phenotype is observed in the case of *ftsh4* mutants [14]. 

Among proteins co-purifying with FTSH4^TRAP.FLAG^ also two stomatin-like proteins (Slp1 and Slp2) from mitochondrial SPFH family were identified. These proteins were implied in the organization of respiratory chain super-complexes in plant mitochondria [30]. Slp1 and Slp2 are homologues of mammalian SLP2 that is involved in mitochondrial fusion and formation of protein complexes in the inner membrane [31,32]. Recent data indicate that human SLP2 plays a role as a membrane scaffold required for the spatial organization of inner membrane proteases such as the FTSH4 counterpart, YME1L [32]. Our data provide an interesting link between FTSH4 and Slp1/Slp2, however it remains to be elucidated whether plant stomatin-like proteins, like their mammalian homolog SLP2, anchor a complex containing *i*-AAA protease in the inner mitochondrial membrane.

Overall, the identification of mitochondrial FTSH4’s potential in vivo interaction partners and substrates gives more a detailed view of molecular functions of this protease in the mitochondria. Conversely, the further, independent validation of selected identified candidates is required.

### 2.2. Identification of Novel Physiological Substrates of FTSH4 

**Figure 3:**
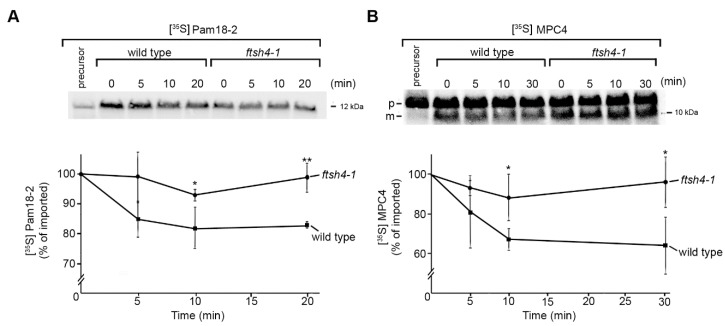
Novel proteolytic substrates of FTSH4 protease. (**A**) Degradation of Pam18-2 by FTSH4 following its in vitro import into mitochondria. Radiolabeled Pam18-2 was imported into mitochondria derived from either wild type or *ftsh4-1* plants. The stability of newly imported precursor upon further incubation at 26 °C was analyzed by SDS-PAGE and autoradiography. Quantification of [^35^S] Pam18-2 in mitochondria is represented in the lower panel. Newly imported Pam18-2 was set to 100%. Data represent mean ± SD of three independent experiments. * *p* < 0.02 and ** *p* < 0.003 (*t*-student test); (**B**) Degradation of MPC4 processed form by FTSH4 following its in vitro import into mitochondria. Radiolabeled MPC4 was imported into mitochondria derived from either wild type or *ftsh4-1* plants. The stability of newly imported MPC4 upon further incubation at 26 °C was analyzed by SDS-PAGE and autoradiography. Quantification of [^35^S] MPC4 processed form in mitochondria is represented in the lower panel. Newly imported MPC4 was set to 100%. Data represent mean ± SD of three independent experiments. * *p* < 0.05 (*t*-student test). m—MPC4 processed form, p—MPC4 precursor.

To assess whether, among proteins co-purifying with FTSH4^TRAP.FLAG^ there are novel proteolytic substrates of this *i*-AAA protease, we analyzed the “in organello” stability of selected proteins. We monitored the kinetics of degradation of candidate proteins inside mitochondria isolated from *ftsh4* mutant and wild type plants. It is expected that the stability of proteolytic substrates will be enhanced in the mitochondria of mutants devoid of functional protease required for their turnover [12,33]. This assay allowed us to identify two novel FTSH4 proteolytic substrates, such as the component of mitochondrial protein import machinery Pam18-2 and the processed form of mitochondrial pyruvate carrier (MPC4). Due to the inaccessibility of specific antibodies, both proteins were synthesized in a cell-free system in the presence of [^35^S] methionine and imported post-translationally into mitochondria isolated from wild type and the *ftsh4* mutant (Figure 3). We found that newly imported Pam18-2 and the processed form of MPC4 were rapidly degraded in wild type mitochondria, but accumulated in *ftsh4* mitochondria, which is consistent with FTSH4-dependent proteolysis. Among tested candidates, there were also proteins (including Slp1, GCD, Mic60) characterized by slow turnover rates already in wild type mitochondria that precluded verification of these proteins in the context of this assay. 

### 2.3. FTSH4 Degrades Mitochondrial Carbonylated Proteins 

**Figure 4:**
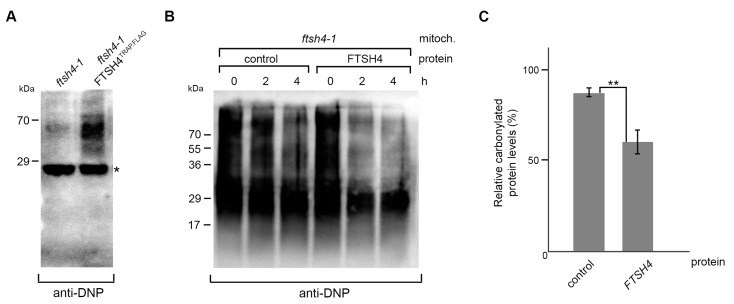
FTSH4 degrades oxidatively damaged mitochondrial proteins. (**A**) Anti-DNP (dinitrophenyl hydrazone) immunoblot detection of carbonylated proteins co-precipitating with FTSH4^TRAP.FLAG^. The cross-reaction of IgG light chain is marked with asterisk. (**B**) The degradation of mitochondrial carbonylated proteins by FTSH4 protease was assessed by immunoblot analysis of the levels of carbonylated proteins in mitochondrial extract from *ftsh4-1* mutant incubated at 35 °C with or without FTSH4 protein. (**C**) Quantification of the remaining carbonylated proteins after 2 h incubation with or without FTSH4 protein, shown in (**B**). For each sample, the amount of carbonylated proteins was set to 100% at time point 0 h. Data represent mean ± SEM of three independent experiments. ** *p* < 0.03 (*t*-student test).

Across species, the lack of *i*-AAA protease is associated with the accumulation of oxidatively damaged proteins in the mitochondria [9,14]. Although it is very probable that these ATP-fueled proteolytic machineries can directly participate in the removal of carbonylated proteins from the mitochondria, so far no evidence has been provided. We found that amongst proteins co-purifying with FTSH4^TRAP.FLAG^ in the substrate trapping assay were oxidatively damaged proteins, suggesting that modification of proteins by reactive oxygen species could trigger their recognition and degradation by the protease (Figure 4A). To test this hypothesis, we performed in vitro analysis employing mature FTSH4 protease produced in the insect-cell lysate [34]. We subjected mitochondrial fractions enriched in carbonylated proteins (derived from *ftsh4* mutant) to FTSH4 or control lysate and analyzed stability of oxidatively modified proteins using Oxiblot detection system. We found that upon FTSH4 addition, the stability of carbonylated proteins is significantly compromised (Figure 4B,C). These data indicate that FTSH4 degrades oxidatively damaged mitochondrial proteins.

## 3. Materials and Methods

### 3.1. Plant Material and Growth Conditions

*Arabidopsis thaliana* lines: ecotype Col-0 wild type, T-DNA insertion lines *ftsh4-1* (SALK_035107/TAIR) [35] and *ftsh4-1* FTSH4^TRAP.FLAG^ (*ftsh4-1* FTSH4(H486Y)) mutant line [12], were grown on 0.5 Murashige and Skoog (MS) medium supplemented with 3% (*w*/*v*) sucrose in chambers in a 16 h light/8 h dark (long day, LD) photoperiod at 22 °C for 2 weeks with a light intensity of 150 μmol m^−2^ s^−1^ [12]. 

### 3.2. Isolation of Mitochondria from Arabidopsis thaliana

Isolation of mitochondria from 14-day-old seedlings was performed as described in [36].

### 3.3. Substrate-Trapping Assay

Mitochondria of FTSH4^TRAP.FLAG^ or control line were resuspended in digitonin solubilization buffer (1% digitonin, 20 mM Tris-HCl, 0.1 mM EDTA, 100 mM NaCl, 10% glycerol, pH 7.7) at 1 mg/mL. PMSF and EDTA-free protease inhibitor cocktail were added and samples were incubated for 30 min at 4 °C with mixing. After clarifying centrifugation, solubilized material was loaded on anti-FLAG affinity matrix and incubated under constant rotation for 1 h 30 min at 4 °C. After excessive washing steps proteins were eluted with Laemmli sample buffer and subjected to SDS–PAGE. Subsequently, protein bands were excised from the gel and analyzed by liquid chromatography coupled to the mass spectrometer in the Laboratory of Mass Spectrometry, Institute of Biochemistry and Biophysics, Polish Academy of Sciences (Warsaw, Poland). Samples were concentrated and desalted on a RP-C18 pre-column (Waters, Milford, MA, USA), and further peptide separation was achieved on a nano-Ultra Performance Liquid Chromatography (UPLC) RP-C18 column (Waters, BEH130 C18 column, 75 µm i.d., 250 mm long) of a nanoACQUITY UPLC system, using a 100 min linear acetonitrile gradient. Column outlet was directly coupled to the Electrospray ionization (ESI) ion source of the Orbitrap Velos type mass spectrometer (Thermo, San Jose, CA, USA), working in the regime of data dependent MS to MS/MS switch. An electrospray voltage of 1.5 kV was used. Raw data files were pre-processed with Mascot Distiller software (version 2.4.2.0, MatrixScience, London, UK). The obtained peptide masses and fragmentation spectra were matched to The Arabidopsis Information Resource (TAIR) database (35,386 sequences/14,482,855 residues) using the Mascot search engine (Mascot Daemon v. 2.4.0, Mascot Server v. 2.4.1, MatrixScience, London, UK). The following search parameters were applied: enzyme specificity was set to trypsin, peptide mass tolerance to ±20 ppm and fragment mass tolerance to ±0.1 Da. The protein mass was left as unrestricted, and mass values as monoisotopic with one missed cleavage being allowed. Alkylation of cysteine by carbamidomethylation as fixed, and oxidation of methionine was set as a variable modification. Protein identification was performed using the Mascot search engine (MatrixScience), with the probability based algorithm. The statistical significance of peptide identifications was estimated using a joined target/decoy database search approach, FDR (False Discovery Rate) was set below 1%. Proteins enriched at least two times in two independent biological replicates over the background samples were considered as co-purifying with FTSH4^TRAP.FLAG^. Protein levels were estimated based on exponentially modified protein abundance index (emPAI) [37,38]. 

### 3.4. Immunoblot Analysis

Protein samples were resolved on SDS-PAGE and transferred on PVDF membrane. Immunodecoration was made with antibodies against AtMic60 [29], Slp1 [30], Tim17-2 [39], Tom40 [40], FLAG-tag (Sigma-Aldrich, Steinheim, Germany, F1804), mtHsp70 (Agrisera, Vännäs, Sweden, AS08 347), cytochrome c (Agrisera, AS08 343A), FTSH4 (Agrisera, AS07 205). Detection of carbonylated proteins was performed using OxiSelect Protein Carbonyl Immunoblot Kit (Cell Biolabs, San Diego, CA, USA) according to the manufacturer’s instructions. Proteins were visualized with enhanced chemiluminescence [41] using a G-BOX ChemiXR5 (Syngene, Cambridge, UK).

### 3.5. Synthesis of Radiolabeled Precursor Proteins

Radiolabeling of selected mitochondrial proteins (mitochondrial pyruvate carrier 4 (MPC4; At4g22310.1) and Pam18-2 (At3g09700.1)) was performed using well-established procedures [42]. Briefly, for in vitro transcription, PCR products containing an SP6 promoter upstream the open reading frame were generated using cDNA as a template. Subsequently, in vitro transcription was carried out according to the manufacturer’s recommendation (mMESSAGE mMACHINE SP6 kit; Ambion, Foster City, CA, USA ), RNA was purified (MEGAclear kit; Ambion, Foster City, CA, USA) and used for in vitro translation in reticulocyte lysate (TNT kit; Promega, Mannheim, Germany) that was supplemented with [^35^S] methionine.

### 3.6. [^35^S]-Labelled Protein Uptake into the Mitochondria and in Organello Degradation Assay

Mitochondrial pre-proteins radiolabeled with [^35^S] methionine were imported into mitochondria of wild type or *ftsh4* mutant according to [42,43]. Briefly, radiolabeled protein uptake into mitochondria was performed at 26 °C in the presence of 2 mM NADH, 5 mM sodium succinate, 2 mM ATP, 2.5 mM methionine, 5 mM creatine phosphate, and 100 µg/mL creatine kinase in import buffer (3% (*w/v*) BSA, 250 mM sucrose, 80 mM KCl, 5 mM MgCl_2_, 10 mM MOPS/KOH, pH 7.2, and 2 mM KH_2_PO_4_). To analyze stability of imported protein import reaction was stopped after 20 min by placing samples on ice and addition of 1 µM valinomycine and 8 µM antimycine. After trypsin digestion (100 μg/mL, 20 min at 4 °C) of non-imported proteins, protease was inhibited by addition of 1.3 μg/μL trypsin inhibitor (5 min at 4 °C). Mitochondria were washed twice in import buffer containing trypsin inhibitor and 2 mM PMSF and resuspended in import buffer supplemented with 2 mM NADH, 5 mM sodium succinate, 2 mM ATP, 2.5 mM methionine, 5 mM creatine phosphate, and 100 µg/mL creatine kinase and 12.5 µM ZnSO_4_. Samples were incubated at 26 °C for the indicated times to allow for proteolysis and subsequently resolved by SDS-PAGE. Radiolabeled proteins were visualized by digital autoradiography (PharosFX Plus Systems, Bio-Rad, Hercules, CA, USA) and analyzed by Quantity One software (Bio-Rad).

### 3.7. Cell-Free Synthesis of FTSH4

For in vitro transcription, PCR product was generated using RTS Linear Template Kit (Biotechrabbit, Hennigsdorf, Germany) and cDNA as a template. Expression of mature FTSH4 (54–717 aa) was performed with *Spodoptera frugiperda*-based cell-free expression system (RTS Insect Membrane Kit, Biotechrabbit) according to the provided manual. 

### 3.8. In Vitro Degradation Assay of Carbonylated Proteins

To assess ability of FTSH4 to degrade carbonylated proteins, the in vitro degradation assay utilizing protease produced in cell-free expression system was performed [44]. Briefly, mitochondria isolated from *ftsh4-1* mutant grown under stress conditions (30 °C, long day (LD) photoperiod) were resuspended in assay buffer (25 mM HEPES/KOH pH 8.0, 1% digitonin, 100 mM KCl, 10 mM MgCl_2_, 1 mM DTT, 10% glycerol, 25 µM ZnSO_4_, 5 mM ATP, 5 mM creatine phosphate, and 100 µg/mL creatine kinase, 2 mM PMSF) with the addition of insect-cell lysate containing FTSH4 or control lysate. Samples were incubated at 35 °C for indicated time points; followed by SDS-PAGE and immunodetection of carbonylated proteins. 

## 4. Conclusions

Here, we provide a list of potential substrates and binding partners of FTSH4 inside mitochondria. Among them, we found two novel proteolytic targets of this plant *i*-AAA protease. Noteworthy, part of the proteins that are designated for FTSH4-dependent removal are oxidatively damaged. Overall, our findings suggest that FTSH4 is linked to respiratory function, protein biogenesis pathways and inner membrane organization. Our data constitute a significant resource for studies addressing the role of plant *i*-AAA in these diverse aspects.

## Figures and Tables

**Table 1:** Proteins co-purifying with FTSH4^TRAP.FLAG^. Listed are all proteins that were specifically co-purifying with proteolytically inactive FTSH4 (proteins enriched at least two times in two independent biological replicates over the background samples (Table S1)). Functional categories and mitochondrial sub-localization were assigned based on the Uniprot database and the literature. AGI—*Arabidopsis* Gene ID.

Functional Category	AGI	Protein	Localization
Transmembrane transport	AT1G25380.1	Nicotinamide adenine dinucleotide transporter 2	inner membrane
	AT4G22310.1	Mitochondrial pyruvate carrier 4 (MPC4)	inner membrane
Metabolism	AT4G35260.1	Isocitrate dehydrogenase NAD regulatory subunit 1	matrix
	AT1G47420.1	Succinate dehydrogenase flavoprotein 5	inner membrane
	AT5G66760.1	Succinate dehydrogenase flavoprotein 1	inner membrane
	AT3G47930.1	l-galactono-1,4-lactone dehydrogenase	intermembrane space
	AT5G23300.1	Dihydroorotate dehydrogenase	inner membrane
Mitochondrial protein import	AT1G53530.1	Mitochondrial inner membrane protease subunit 1	inner membrane
	AT2G46470.1	OXA1-like protein	inner membrane
	AT3G09700.1	Pam18-2	inner membrane
	AT5G05520.1	Sam50-2	outer membrane
	AT2G35795.1	Pam18-1	inner membrane
	AT2G37410.1	Tim17-2	inner membrane
Membrane organization	AT5G54100.1	Stomatin-like protein 2 (Slp2)	inner membrane
	AT4G27585.1	Stomatin-like protein 1 (Slp1)	inner membrane
	AT4G39690.1	Mic60	inner membrane
Unknown	AT5G62270.1	Gamete cell defective (GCD)	unknown

## Supplementary Materials

Supplementary materials can be found at www.mdpi.com/1422-0067/18/11/2455/s1.
